# A Multidisciplinary Research Agenda for Understanding Vaccine-Related Decisions

**DOI:** 10.3390/vaccines1030293

**Published:** 2013-07-18

**Authors:** Heidi Larson, Julie Leask, Sian Aggett, Nick Sevdalis, Angus Thomson

**Affiliations:** 1 London School of Hygiene & Tropical Medicine, London WC1 7HT, UK; E-Mail: heidi.larson@lshtm.ac.uk; 2 School of Public Health, University of Sydney, Sydney NSW 2006, Australia; 3 International Engagement Project Manager, The Wellcome Trust, London NW1 2BE, UK; E-Mail: s.aggett@wellcome.ac.uk; 4 Center for Patient Safety and Service Quality and Department of Surgery and Cancer, Imperial College London, London W21 PG, UK; E-Mail: n.sevdalis@imperial.ac.uk; 5 Vaccination Advocacy, Sanofi Pasteur, Lyon 69007, France; E-Mail: angus.thomson@sanofipasteur.com

**Keywords:** vaccination, policy, communication, attitudes

## Abstract

There is increasingly broad global recognition of the need to better understand determinants of vaccine acceptance. Fifteen social science, communication, health, and medical professionals (the “Motors of Trust in Vaccination” (MOTIV) think tank) explored factors relating to vaccination decision-making as a step to building a multidisciplinary research agenda. One hundred and forty seven factors impacting decisions made by consumers, professionals, and policy makers on vaccine acceptance, delay, or refusal were identified and grouped into three major categories: cognition and decision-making; groups and social norms; and communication and engagement. These factors should help frame a multidisciplinary research agenda to build an evidence base on the determinants of vaccine acceptance to inform the development of interventions and vaccination policies.

## 1. Introduction

Immunization has saved millions of lives worldwide since the introduction of the first vaccine more than 200 years ago. However, the sustained success of immunization programs, a cornerstone of public health, is challenged by increasing vaccine questioning, hesitancy, and refusals. These occur for a range of reasons varying from religious and philosophical to concerns about vaccine safety and schedules, or questions about the relevance of some vaccines [1,2]. Moreover, for each vaccine and its target disease(s) there is a unique set of interested “publics” with different positive and negative perceptions and attitudes to vaccination. High profile vaccine scares have brought significant disruption or cessation to entire vaccine programs. For example, despite Andrew Wakefield’s 1998 article in *The Lancet* [3] being refuted, retracted, and declared fraudulent [4], uptake of measles, mumps and rubella (MMR) vaccination dipped in the UK from 91% in 1998 to 80% by 2004 [5]. There have since been several outbreaks of measles and, 14 years after the local transmission of measles was halted in the UK, the disease was once again reported to be endemic in 2008 [6], and the beginning of 2013 saw the highest rates of measles in two decades. In both 2010 and 2011, there were over 30,000 cases annually of measles in the European region [7]. In another instance, in 2003 five states in Northern Nigeria ordered the boycott of the oral polio vaccine (OPV), alleging that the vaccines were contaminated with anti-fertility substances in a plot by Western governments to reduce the Muslim population [8]. As a result of the boycott, polio reappeared in more than 15 formerly polio-free African countries (and as far afield as Indonesia) [9], and challenges to eradication persist [10]. A more recent example of widespread vaccine refusals was during the 2009–2010 response to the (A)H1N1 pandemic threats, during which populations, including health professionals, around the world had dismally low acceptance of the Influenza A (H1N1) vaccine for a complex mix of reasons from perception of low-to-no disease risk, suspicions around the motives of government and the pharmaceutical industry, and historic memories of reports of an earlier swine flu vaccine causing Guillain–Barré syndrome (GBS). These examples highlight the complex social, historical, political, and power dimensions that influence vaccination uptake [1].

These experiences and the research on drivers of vaccination behavior have shown that vaccination decision-making is driven by different factors according to individual or group experiences and contexts, beliefs, and knowledge. Dependent on the viewpoint of the public/s, healthcare professional or government/healthcare system, the spectrum of attitudes toward vaccines ranges from considering them to be life-saving, to viewing them as a danger to health. Research also demonstrates that facts only go so far in determining decision-making; cognitive heuristics are equally important drivers. For example, the regret that people associate with potential adverse events after MMR vaccination has been shown to be a key predictor of MMR uptake [11]. Other studies have shown that many factors that affect behavior are unrelated to facts or awareness, and that traditional modes of health education that are more message-driven rather than dialog-promoting, may have only a small impact on behavior [12,13]. A recent systematic review of the evidence for effective national immunization schedule promotional communications found no evidence that improved knowledge led to increased childhood vaccine uptake, or even intention to vaccinate [14]. 

To date, much of the literature on vaccination decision-making has identified attitudinal and demographic correlates of complete and incomplete vaccine uptake largely in individuals. The published research has mostly been uni-disciplinary—*i.e*., drawing from a single specialty (like psychology, or sociology, or public health). While important, the field needs to draw from a range of rich theoretical understandings in other areas of health that can inform more holistic frameworks to understand vaccine decisions and their motivations. Multi-disciplinary approaches to understanding vaccination behavior could also further extend the evidence base, making the most of the tools and frameworks available within the different disciplines. Finally, further development of the field will require the design and evaluation of theory, and evidence-informed interventions at an individual, community, and national level to address the identified influences of vaccination decisions.

This paper reports on a two-day workshop with a multidisciplinary group of experts aimed at mapping, firstly, the known and potential drivers and barriers to vaccination at the individual and societal level, and, secondly, a research framework that allows future research to address them. Our ultimate aim was to inform a new research agenda from a rich multidisciplinary evidence base to inform vaccination program design and policy making. 

## 2. Methods

In December 2010, an international think tank called “MOTIV” (Motors of Trust in Vaccination) was convened in London. The think tank deliberately assembled professionals with diverse expertise both within, and beyond, vaccinology. The 15 participants were variously expert in medical science, vaccinology, epidemiology, pediatrics, immunization policy and programs, immunization behavior, global health, psychology, anthropology, sociology, decision science, communication science, advocacy, public engagement, and manufacturing. The specific aim was to map the complex web of factors that may influence decision-making about vaccines at all levels, including individuals, peer groups, clinicians, and policy makers. 

These aims were addressed through a range of interactive sessions. First, a structured brainstorm was carried out where members were asked to spontaneously identify factors affecting vaccination-related behavior—including vaccine acceptance, hesitancy, and refusal—by consumers, professionals, and policy makers. This involved the use of a “reverse brainstorm” technique to help participants look at issues around vaccination uptake “through new eyes”. Here, participants were asked to consider the issue of vaccination from the opposition point of view—and think of ways to make uptake of a vaccine program as *poor* as possible (in this case a fictitious new vaccine with data from clinical trials showing acceptable levels of safety and efficacy). 

Following the reverse brainstorm, participants identified factors/determinants of vaccine decision-making. The ideas were captured in an iterative manner and clustered by MOTIV participants into three major domains. Participants were then assigned to three teams that would each explore one of the major domains. Each team reviewed the list of factors/determinants in their assigned domain and then ranked them based on their expert perceptions according to importance, level of evidence, feasibility/actionability, and the need for more research. These key factors/determinants were distilled into research questions that could be taken forward for further investigation. Key factors/determinants for which a research framework could be developed were finally identified and discussed within the entire MOTIV group. 

## 3. Results

The brainstorming identified poor communication, safety concerns, political issues, anti-vaccination activism, and animal rights as the major areas under which a vaccine program might be derailed. 

Regarding determinants of vaccination decision-making, the MOTIV expert group identified 61 factors that may affect vaccination-related behavior in consumers, professionals and policy makers (Appendix 1). These factors were further iteratively organized into “clusters”, which were grouped under three major domains: cognition and decision-making; groups and social norms; and communications and engagement. 

Research questions for further investigation were derived across the three major domains of influence on vaccination uptake, as detailed below. Boxes 1 and 2 illustrate two exemplars of such research questions.

**Box 1. Which cognitive processes mediate vaccine decision-making and what are their relative roles in different contexts?**

A range of cognitions influence vaccination decision-making, including heuristics. These are cognitive shortcuts used for making decisions about risk. One well-described heuristic in vaccination decisions is omission bias. This bias occurs if poor outcomes arising from an “action” (e.g., a reaction to a vaccine arising from deliberate acceptance of a vaccine) are viewed more unfavorably than poor outcomes arising from an “inaction” (e.g., disease contraction arising from “taking a chance with fate”), even if those outcomes are objectively identical, or omission is in fact more risky. Psychologically, omission bias has been linked to the emotion of regret: decision-makers tend to experience more regret for an outcome that they perceive as a consequence of their own voluntary “action” than for the very same outcome if that is perceived to be a consequence of luck or fate. Within the context of immunization, typically immunizing is seen as a conscious “action” whereas not immunizing (*i.e*., “doing nothing”) is seen as “inaction”. Evidence for omission bias has been demonstrated in relation to pertussis [11,12], MMR [13] and H1N1 vaccines [14].

**Figure 1:**
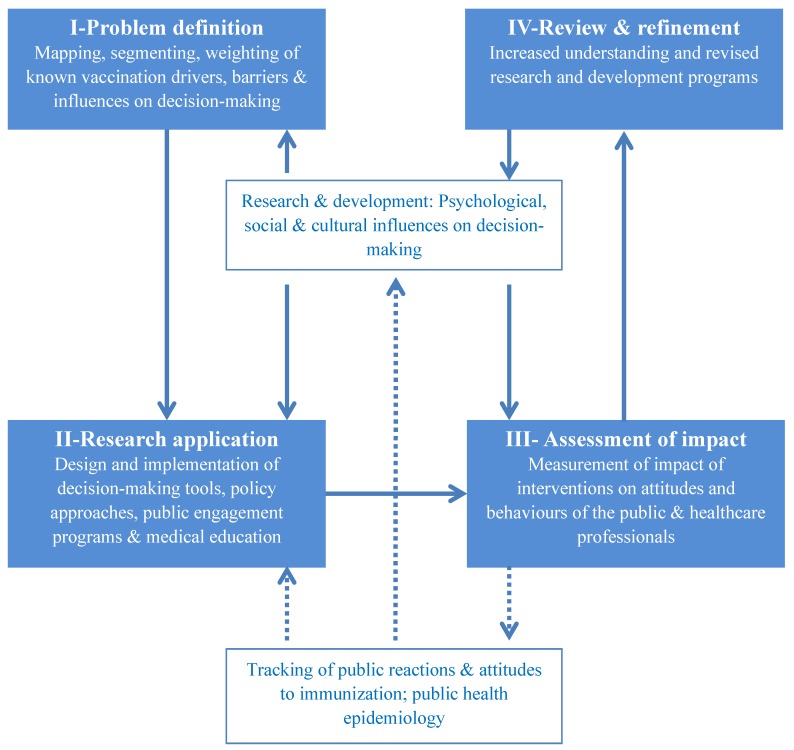
A dynamic multidisciplinary research framework to drive evidence-based policy making in vaccination.

Following the identification of drivers and barriers to vaccination and the related decision making processes, we sought to outline a viable research framework. Figure 1 presents an overarching framework aimed at outlining and systematizing a multidisciplinary research approach that is directly linked to evidence-based policy making. The framework is grounded on the types of themes that emerged through the expert brainstorming—namely the cognitive, social/interpersonal, and communication-related influences on attitudes to immunization and vaccination decision-making. A four-step iterative cycle is described. In the first step, descriptive and experimental research offers scientific definitions and illustrations of the issues to be tackled (e.g., omission bias in immunization decisions). In the second step, the findings are translated into interventions—typically including individual decision-makers (e.g., a de-biasing technique to be applied by community nurses or physicians offering vaccinations), the wider public, and also healthcare professionals. In the third step, these interventions are prospectively evaluated for effectiveness and the findings are fed back into the evidence base in the fourth and final step.

**Box 2. How does engagement with the various publics influence the level of trust in vaccines, vaccinations and vaccination-promoting groups or organizations?**

Which public engagement strategies within the areas of vaccination decision-making and broader healthcare have achieved their goals, and how and why have they achieved their goals? How does/should communication and engagement change according to culture, geographical region or broadcast channel?

Public engagement is an umbrella term for a range of activities that occur at the interface between the specialist and non-specialist. Engagement is defined more by its ethos than by the vehicles of engagement. A key consideration is power: who is driving an engagement process, who owns the conversation, and how far can this process meet different stakeholders’ multiple agendas? The emphasis of engagement is not to get public buy-in for a health program or technology; it is something more collaborative than lobbying or campaigning and goes beyond health promotion. Engagement aims to catalyze a two-way interaction, and well-executed public engagement will ultimately enable more critically aware, insightful decisions for all parties. This may be breaking new and difficult ground for many professionals and scientists and is therefore an important area for future research.

Importantly, the framework rests on robust multidisciplinary research and development process—which includes key social and behavioral sciences. It also identifies the need for real-life longitudinal tracking not only of coverage and disease outbreaks via epidemiological methods, but also of behavioral and social reactions to immunization. The latter are aimed as explanations for and predictors of the former. 

The basic premise of the framework is a dynamic approach to the generation and evaluation of new evidence to drive policy-making and program design. Vaccine decision-making is recognized here as a dynamic field of enquiry that can be rapidly affected by new vaccine developments, novel social movements (e.g., newly emerging social networks) and the increasing quest for evidence-based policy. The iterative link described here between multidisciplinary science and real-life coverage/attitudes to vaccines allows this framework to offer insights into policy making. 

## 4. Discussion

The MOTIV workshop brought together a multidisciplinary group of experts and aimed at mapping drivers and barriers to vaccination to inform future research priorities. The ultimate aim of the workshop was to contribute to a contemporary research agenda, which will in turn inform vaccination policy-making and program design (in the manner outlined in Figure 1). It is clear from the factors identified that public engagement around vaccines needs to be broad and multifactorial, with engagement at multiple levels. These include policy-making (e.g., deliberative democracy), program design (including delivery) and the development of risk communication strategies. 

Methodological improvements are required for better understanding of vaccine decision-making across populations and contexts and over time. Self-reported vaccine uptake and cross-sectional studies (where we assume causation between a certain attitude and behavior from measurements made only at one time point) limit the robustness of research into vaccination decision-making. In this context, attention to improving research design and data quality is essential, to provide a clear understanding of the relative contribution of factors such as trust, risk perception, online networks, peer networks, and misinformation. 

Theoretically sound research frameworks and validated methods are also important. Use of recent, robust evidence-based attitude measurement instruments to evaluate the predictors of MMR uptake clearly shows that differences exist in the way vaccine-acceptors and vaccine-decliners think about several key factors regarding vaccination and disease control [15]. 

The MOTIV approach has some limitations. The faculty consisted of experts across diverse fields but did not exhaust the range of potentially relevant areas of expertise. Moreover, during the group sessions it was agreed that the domain “Communication” is too broad an area and more specific research topics need to be defined within the broader realm of communication. Additionally there was a significant degree of crossover between the domains—for example “Trust” overlaps with “Public engagement”. The question of “*What influences policy decision-making?*” was identified as missing and was subsequently added to the decision-making category. Further, no formal consensus building methods were applied, as the idea-generation techniques used throughout the workshop were solely qualitative.

These limitations notwithstanding, we take the view that the questions outlined, and the proposed framework, are timely. Recent global events have demonstrated a desire for strategic attention to vaccine decision-making. The need to strengthen public support for vaccination efforts is one of the four components that comprise the Global Vaccine Action Plan, endorsed by the World Health Assembly (2012), and catalyzed by the Decade of Vaccines collaboration [13]. Additionally, WHO Strategic Group of Experts (SAGE) on immunization created a working group to specifically address vaccination hesitancy in 2012. 

Just as vaccine development and testing is informed by science and research, so must our understanding of vaccine decision-making by publics, professionals, and policy makers be informed by robust scientific methods. This understanding will more credibly inform appropriate interventions to support decision-making. This framework provides the groundwork for a more explicitly articulated research agenda on vaccine decision-making. It suggests cross-disciplinary investigations (e.g., applying social networking theories to understanding community influence) and provides a starting point for researchers to identify areas well understood and those needing further enquiry. 

An approach to researching vaccine decision-making and to translating the research findings into usable building blocks for policy making has been described, involving a range of multidisciplinary factors that cannot be addressed simply with existing health metrics or by one discipline alone. 

## 5. Conclusions

The aim of the MOTIV think tank was to map what we do and do not know about the drivers of vaccination decision-making, and to look beyond the traditional “one-way” approach to health information. In-depth understanding of complex decision-making processes—through appropriate collaborative research across multiple disciplines—is key to better understanding the drivers and barriers of trust in vaccination, and defining how best to engage publics. This provides a first step towards building a dynamic multidisciplinary research network, in both developed and developing countries, that can synthesize research findings within a coordinated research program, develop interventions, and eventually facilitate increasingly evidence-based vaccination policy. 

## Appendix

**Appendix 1:** Factors (Drivers and Barriers) Affecting Vaccination Uptake and Sample Research Questions.

Domain	Cluster	Potentially influencing factors	Sample research questions
*Cognition and decision-making*	Trust	Trust in national institutions Trust in healthcare workers Trust in authorities/experts Trust in research Trust in policy makers/accountability Trust in media Trust in official information Trust in vaccine industry Trust in vaccines Interpersonal trust Industry-policy maker-researcher trust Transparency Competency	-How should trust be measured? What are the drivers of trust (intellectual, personalities, environmental—external physical drivers, *etc*.)?—Who do different groups trust? Who are real influencers? -Do different publics trust the media differently? -What is the trust spectrum for vaccines? -Who influences different population segments (e.g., mapping trust networks)? -What determines trust in a) health officials and experts; b) healthcare workers; and c) policy makers? -How can public confidence in vaccination be engendered, protected and measured?
	Risk and perception	14. Heuristics (e.g., confirmatory bias, regret bias, omission bias) 15. *Post-hoc* rationalisation 16. Risk-benefit assessments 17. Scientific/medical literacy 18. Physical sensations 19. Perceptions about the faith and fear of injecting substances 20. Perceptions regarding vaccine adverse events, including their likelihood and severity 21. Invisible disease (low perception of risk)	-Which heuristics are most prevalent or widely used when it comes to vaccine decision-making? -How do you change an individual’s norm or reference point? -How multiple heuristics interact in vaccine decision-making? Why are some issues very vaccine-specific whereas others concern several vaccines? -Do actions or thoughts/feelings come first? What is the impact of fear? -Can we create a segmentation of different decision-making styles How do people interpret messages (mental shortcuts, biases, heuristics) and overestimate risks? -Do healthcare workers decide differently for patients vs. themselves? If so, what influences this? -Do healthcare workers’ perceptions mirror those seen in public? Are healthcare workers’ perceptions different from those of the public? -Why do healthcare workers not accept vaccines themselves—even if they recommend them to their patients? Can we measure “what” and “how” people understand and remember (information, experiences)?
*Cognition and decision-making*	*Decision context*	22. Competing priorities; Practical barriers to access 23. Weighting of the factors may change depending on the context 24. Multi-factorial equation involving the individual and groups (institution, culture, religion, social network) 25. Experience of adverse events and how they are managed 26. Safety research on vaccines (at an individual level 27. Mandatory vaccination transfers decision-making away from people and leads to opposition and controversies 28. Level of education/literacy/ scientific and health literacy	-For which segments is having a vaccine a decision? -What makes vaccines a lower priority? -Why are some issues very vaccine-specific whereas others concern several vaccines? -Which public and individual contexts influence vaccine decision-making? -What is the relationship between decision-making and notions of climate change? How can healthcare systems adapt to different public attitudes (policy through to implementation)? Why do some controversies remain local or specific to one particular vaccine? Macro perspectives are needed to link behavior to the broader context (e.g., government) within a theoretical framework To what extent do vaccine-critical groups influence decision-making? Poor literacy leads to low uptake but good literacy also leads to low uptake. Can we solve the puzzle? Who are the policy influencers?
*Groups and social norms*	*Groups/grouping*	29. Social networks 30. Religious groups 31. Groups by education-level 32. Alternative medicine groups 33. Political groups 34. Anti-vaccination groups 35. Family groups/structures 36. Patient groups 37. Consumer groups 38. Professional groups 39. Peer pressure 40. Heterogeneous groups 41. Demographic (socioeconomic) groups 42. Influence of alternative healthcare givers such as Chinese medicine	What is the role of parental questioning about health and education and the shift towards ‘intensive’ parenting? Role of peer pressure? How do memes (a unit of cultural information transferable from one mind to another) travel between groups in a constantly and globally connected society? What is the role of culture and religion on decision-making? How do a few anti-vaccine individuals influence the ‘swing voters’/vaccine hesitants? Understand the heterogeneity of groups/publics Role of social pressure and altruism in decision-making. What are the benefits to society?
*Groups and social norms*	*Groups/grouping*		Define the network of influencers and the context. What is the best way to communicate with this network? Can we develop a new model to measure and/or assess the level of influence? What are the factors that make a family vaccination strategy (e.g., “cocoon” strategy in pertussis) “to protect your family members” successful or not? Can we learn lessons from military strategy about engaging with publics, especially with respect to parallel access and trust building and “hearts and minds” campaigns? Is there a way to better understand and/or to classify vaccine-critical groups? Is there a real/organised anti-vaccine lobby?
	*Social norms*	43. Social and cultural norms 44. Vaccination as a routine/norm	-Which social norms influence vaccination decisions? -How can acceptance of safe, effective vaccines be sustained as a social norm? -How do different types of protective norms influence decision-making?
*Communications* *and engagement*	*Sender*	45. Healthcare workers communication skills 46. Physicians poor vaccine education 47. Media 48. The authority of the messenger impacts the credibility of the source of the information 49. Tone in communication 50. Ensuring that people feel “listened to” 51. Level of how informed and educated (content of training) the media and journalist are 52. Health care workers level of confidence in providing information to their patients 53. Healthcare worker temperament	How do physicians communicate with patients? How do patients communicate with physicians? What are the outcomes of these interactions? What is the impact of fear? How should messages be framed or constructed? How does the use of exemplars and narratives apply to vaccination? How do ideas travel and how do we communicate with each other in the “connected generation”? What does “constantly connected” mean? Is Institute of Medicine report on consumer-provider relationship regarding vaccines globally relevant? What are the sources that journalists use for information? How credible are these sources?
